# A Robust Memristor-Enhanced Polynomial Hyper-Chaotic Map and Its Multi-Channel Image Encryption Application

**DOI:** 10.3390/mi14112090

**Published:** 2023-11-12

**Authors:** Kun Qian, Yang Xiao, Yinjie Wei, Di Liu, Quanwen Wang, Wei Feng

**Affiliations:** 1 Key Laboratory of Hunan Province on Information Photonics and Freespace Optical Communications, Hunan Institute of Science and Technology, Yueyang 414006, China; tsienkun@hnist.edu.cn; 2 School of Physics and Electronic Science, Hunan Institute of Science and Technology, Yueyang 414006, China; 3 School of Mathematics and Computer Science, Panzhihua University, Panzhihua 617000, China; yangx0209@126.com (Y.X.); weiyinjie2024@126.com (Y.W.); liudi200105@126.com (D.L.); quanwenwang@pzhu.edu.cn (Q.W.)

**Keywords:** memristor, hyper-chaotic map, image encryption, multi-channel, security analysis

## Abstract

Nowadays, the utilization of memristors to enhance the dynamical properties of chaotic systems has become a popular research topic. In this paper, we present the design of a novel 2D memristor-enhanced polynomial hyper-chaotic map (2D-MPHM) by utilizing the cross-coupling of two TiO${}_{2}$ memristors. The dynamical properties of the 2D-MPHM were investigated using Lyapunov exponents, bifurcation diagrams, and trajectory diagrams. Additionally, Kolmogorov entropy and sample entropy were also employed to evaluate the complexity of the 2D-MPHM. Numerical analysis has demonstrated the superiority of the 2D-MPHM. Subsequently, the proposed 2D-MPHM was applied to a multi-channel image encryption algorithm (MIEA-MPHM) whose excellent security was demonstrated by key space, key sensitivity, plaintext sensitivity, information entropy, pixel distribution, correlation analysis, and robustness analysis. Finally, the encryption efficiency of the MIEA-MPHM was evaluated via numerous encryption efficiency tests. These tests demonstrate that the MIEA-MPHM not only possesses excellent security but also offers significant efficiency advantages, boasting an average encryption rate of up to 87.2798 Mbps.

## 1. Introduction

In 1971, Professor Chua [1] first proposed the concept of the memristor. Its expression can be defined by the relationship between charge and magnetic flux. Compared to the three fundamental circuit components, resistors, capacitors, and inductors, memristors possess unique memory resistance functional characteristics. Due to this distinctive memory property, memristors can find applications in various fields, including intelligent computing [2,3], neural networks [4,5,6], data mining [7], memory devices [8,9,10], and chaotic circuits [11,12,13], among others. However, because memristors have not been physically realized in the real world, research on memristors and memristor circuits has not attracted the attention of researchers. The situation remained unchanged until 2008, when HP Labs successfully developed a physical memristor using nanomaterials [14], thereby sparking a surge of research interest in memristors.

The latest research has discovered that the introduction of memristors into certain chaotic maps, such as sine maps [15], Hénon maps [16], and higher-order chaotic maps [12], can enhance the complexity of these maps by generating more diverse dynamical behaviors. Due to the introduction of memristors, new memristor-enhanced chaotic systems can possess one or more sets of equilibrium points. Simultaneously, their dynamical characteristics are not only sensitive to parameters but also strongly dependent on the initial values of the memristors. Therefore, the dynamical characteristics of these new chaotic systems are more complex than those of the original chaotic systems. They exhibit various complex dynamical behaviors, including hyper-chaos, transient chaos, coexisting attractors, multistable states, and state transitions. In [17], Bao et al. proposed a second-order chaotic map model based on a memristor, which has infinite unstable and critical stable fixed points and exhibits hyper-chaotic behavior. In [18], Fang et al. used memristors with coexisting hysteresis curves and dual local active regions to replace diodes in Chua’s circuit, and obtained a multi-scroll fourth-order hyper-chaotic system. In [19], Peng et al. used Caputo fractional-order difference to construct a map model based on a second-order memristor, which shows rich dynamical behaviors in the form of fractional order. Once the aforementioned research was reported, it immediately garnered significant attention from numerous researchers. Subsequently, an increasing amount of related research has emerged on the utilization of memristors to enhance chaotic map [20,21,22,23,24].

In 2014, Professor Chua proposed that local activity is the origin of complexity [25]. Complex behaviors and rich dynamics may arise only in locally active systems. Some studies have shown that materials such as NbO${}_{x}$, TaO${}_{x}$, TiO${}_{x}$, and VO${}_{2}$ can be used to manufacture memristors [26] and exhibit local active characteristics [27]. The DC voltage–current (V-I) characteristics of a local active memristor have a negative slope region. Therefore, circuit systems constructed with local active memristors can generate more complex and diverse dynamic behaviors. Compared to original chaotic systems, memristor-enhanced chaotic systems are more suitable for applications in security fields, such as image encryption [28,29,30,31,32]. Accordingly, the application of memristor-enhanced chaotic systems in the field of image encryption has become a research hotspot. Lin et al. [29] proposed Chua’s chaotic system composed of piecewise linear (PWL) memristors and utilized it for image encryption. This encryption scheme possesses a larger key space and provides robust security. Li et al. [30] used a third-order magnetron memristor device to construct a chaotic circuit and conducted circuit experiments. The system has rich dynamical behaviors such as antisymmetry, multistable states, and transient chaos. In addition, based on this memristive chaotic system, they designed a chaotic image encryption scheme based on dynamic deoxyribonucleic acid (DNA) operations and dynamic diffusion. Experimental results show that the image encryption system has high security and good anti-attack capabilities. Chen et al. [31] constructed a new 4D hyper-chaotic system based on a magnetic-controlled memristor model that exhibits rich dynamical behaviors. The simulation results indicate that the system can generate complex chaotic attractors. Subsequently, they designed an image encryption scheme based on this system, and further evaluated the performance of the encryption scheme through tests such as key space and correlation analysis. Ye et al. [32] designed a new discrete memristive chaotic system using multiple memristors. They compared this discrete memristive chaotic system with other classical discrete chaotic systems. The comparison results indicate that the chaotic interval of their new system is larger. Consequently, they designed a new encryption algorithm based on this chaotic system and evaluated its security using indicators such as pixel distribution and pixel correlation.

At present, cryptanalysis research on chaotic image encryption shows that some existing chaotic image encryption schemes still have some security, practicality, and efficiency problems [33,34,35,36,37]. For example, the chaotic interval of the system used is narrow, the chaotic interval is discontinuous, the system trajectory distribution is uneven, etc. It is worth noting that existing chaotic image encryption algorithms generally suffer from low encryption efficiency [38,39,40,41,42]. Therefore, in this paper, we first constructed a 2D memristor-enhanced polynomial hyper-chaotic map (2D-MPHM). Then, based on this hyper-chaotic map, we further designed a multi-channel image encryption algorithm (MIEA-MPHM). Overall, the work presented in this paper has the following innovations and contributions:

(1) We utilized two memristors to construct a 2D discrete polynomial hyper-chaotic map, known as a 2D-MPHM, in a cross-coupled arrangement. Relevant tests and analyses reveal that this hyper-chaotic map possesses various advantages, including a wide chaotic range, uniformly distributed trajectories, high complexity, and excellent randomness.
(2) With our proposed 2D-MPHM, a novel multi-channel image encryption algorithm called an MIEA-MPHM was devised, which incorporates seven encryption steps, namely the generation of an initial chaotic sequence, multi-channel pixel fusion, generation of image-specific keystreams, and two rounds of double-vector (column or row) staggered diffusion and full-range pixel scrambling.
(3) Extensive tests and analyses have confirmed that our proposed MIEA-MPHM not only offers exceptionally high security but also demonstrates significant efficiency advantages, boasting an average encryption rate of up to 87.2798 Mbps.

The remaining sections of our work are structured in the following manner: Section 2 introduces the 2D-MPHM and evaluates its performance by employing some chaotic performance indicators; in Section 3, there is a detailed description of both the overall framework of the MIEA-MPHM and the individual encryption steps employed; Section 4 presents a range of tests and analyses aiming to prove and emphasize the superior security and efficiency of the MIEA-MPHM; and our work is concluded in the last section.

## 2. Proposed 2D-MPHM

This section provides a detailed introduction to our proposed discrete hyper-chaotic map called a 2D-MPHM, which is constructed using the HP memristor model. This hyper-chaotic map is then assessed and analyzed using several chaotic indicators.

### 2.1. Construction of 2D-MPHM

According to the ion drift model of the classic HP TiO${}_{2}$ memristor, its memristance M(q) is expressed as

(1) $$M\left(q\right)=R_{OFF}\left(1-\frac{\mu_{V}R_{ON}}{D^{2}}q\left(t\right)\right),$$

where $\mu_{V}$ represents the migration rate of doped ions, *D* denotes the thickness of the semiconductor film of the memory resistor, and $R_{ON}$ and $R_{OFF}$ are the resistance values of the memristor when it is in the low-resistance state and high-resistance state, respectively. These values are determined by the physical properties of the memristor. q(t) is the amount of charge passing through the memristor, which is equal to the integral of current intensity over time. Specifically,

(2) $$q\left(t\right)=k_{m}\int_{-\infty }^{t}i(t)dt=q_{0}\left(t\right)+k_{m}\int_{t_{0}}^{t}i(t)dt,$$

where $k_{m}$ is a constant, typically taken as 1. The voltage V(t) between the two ports of the memristor can be represented as

(3) $$V\left(t\right)=M\left(q\right)i\left(t\right)=\left(a_{m}-b_{m}\left(q_{0}+k_{m}\int_{t_{0}}^{t}i(t)dt\right)\right)i\left(t\right).$$

**Figure 1:**
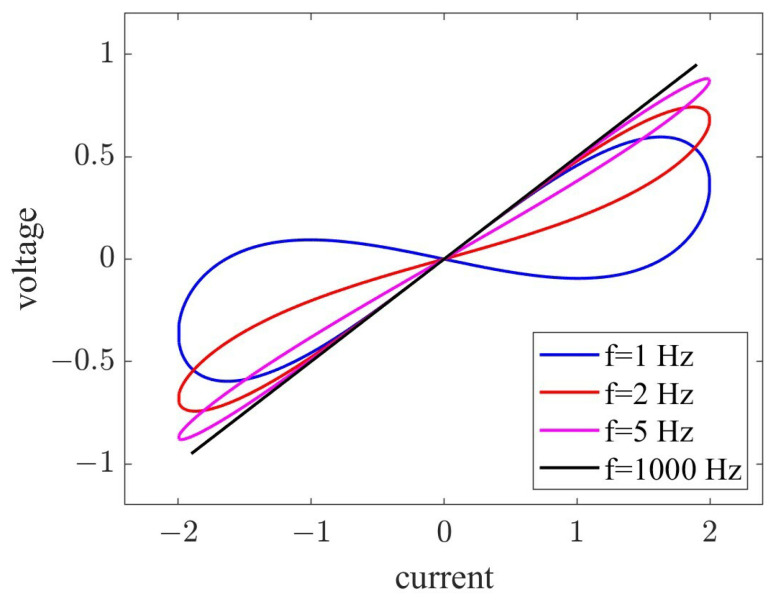
Pinched hysteresis loops of adopted memristor model.

According to the discrete memristor model proposed by Peng et al. [12], the discrete voltage V(n) of the TiO${}_{2}$ memristor can be represented as

(4) $$V\left(n\right)=\left(a_{m}-b_{m}\left(q_{0}+k_{m}\sum_{j=0}^{n}i_{j}\right)\right)i_{n},$$

where $a_{m}=R_{OFF}$ and $b_{m}=R_{OFF}\mu_{V}R_{ON}/D^{2}$ represent constants related to memristors. Generally, $R_{OFF}\;\gg \;R_{ON}$. In this study, we set $\left\{a_{m},b_{m},k_{m},q_{0}\right\}$ as {1,3,1,0.1}. For the discrete memristor model described above, when a sinusoidal excitation signal $i_{n}=A\sin\left(2\pi fn\right)$ is applied, one can observe a pinched hysteresis loop of the memristor, as depicted in Figure 1. As one can see, when the frequency *f* of sinusoidal signal $i_{n}$ increases, the memristor’s pinched hysteresis loop becomes narrower. As *f* tends toward infinity, the pinched hysteresis loop contracts into a straight line. These characteristics demonstrate that our adopted discrete memristor model satisfies the definition of a memristor.

**Figure 2:**
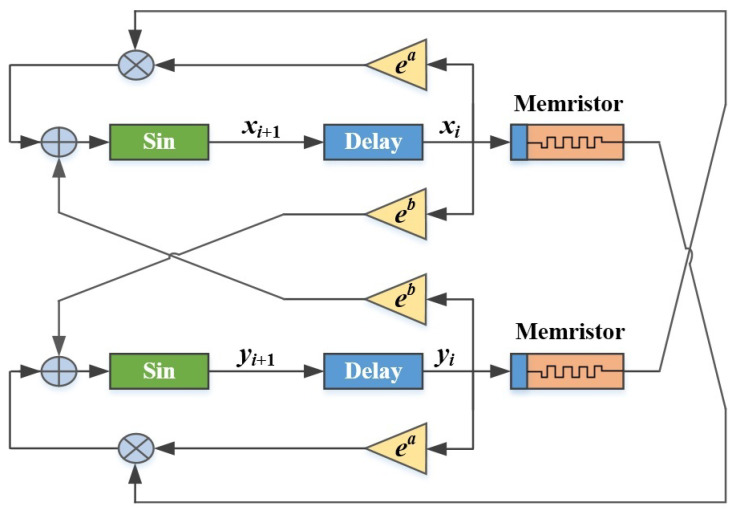
Coupling schematic diagram for 2D-MPHM.

In our study, this discrete memristor model is exploited to enhance the chaotic and complex properties of a 2D polynomial map. The coupling schematic diagram for the 2D polynomial map and two memristors is shown in Figure 2.

As can be seen, the two discrete memristors are applied to the system state values $x_{i}$ and $y_{i}$ of the polynomial map, respectively, and then coupled to $x_{i+1}$ and $y_{i+1}$ in a cross-over manner. Moreover, two system control parameters *a* and *b* are set on the exponent with *e* as the base to speed up the divergence of the system state values. Finally, the sine function is used to constrain the system state values to the range of [−1,1]. In this way, we eventually obtained a new memristor-enhanced polynomial map called a 2D-MPHM. Specifically, the definition of the 2D-MPHM is as follows:

(5) $$\left\{\begin{array}{c} x_{i+1}=\sin\left(e^{a}x_{i}\left(a_{m}-b_{m}\left(q_{0}+k_{m}\sum_{n=1}^{i}y_{n}\right)\right)+e^{b}y_{i}\right),\\ y_{i+1}=\sin\left(e^{a}y_{i}\left(a_{m}-b_{m}\left(q_{0}+k_{m}\sum_{n=1}^{i}x_{n}\right)\right)+e^{b}x_{i}\right), \end{array}\right.$$

where *a* and *b* are the control parameters of the 2D-MPHM, and $a_{m}$, $b_{m}$, $k_{m}$, and $q_{0}$ are the parameters of the adopted memristor model.

### 2.2. Lyapunov Exponent

**Figure 3:**
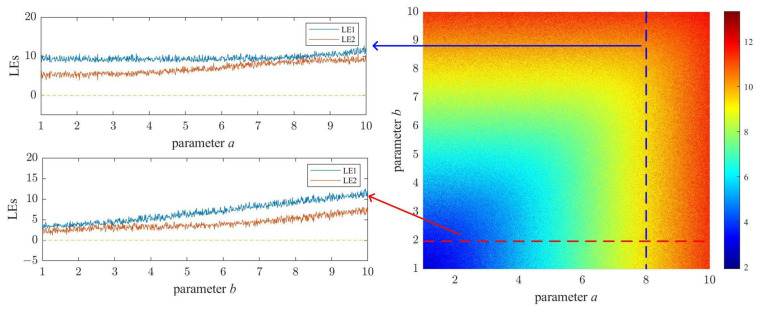
LE diagram of 2D-MPHM.

In related research on chaotic systems, the Lyapunov exponent (LE) is commonly utilized to assess the chaotic characteristics of such systems. A system is considered chaotic if it possesses one or more positive LEs. Furthermore, if a system exhibits two or more positive LEs, it signifies that the system is a hyper-chaotic system. Figure 3 shows the LE diagram of the 2D-MPHM. The LEs of the 2D-MPHM were calculated using the QR decomposition method [43]. Both parameters *a* and *b* were scanned from 1 to 10 with a step size of 0.01.

Upon observing Figure 3, it is evident that within the provided parameter range, the two LEs owned by the 2D-MPHM are consistently positive. Consequently, it can be concluded that the 2D-MPHM is a hyper-chaotic map. Moreover, the maximum LE of the 2D-MPHM increases as the values of *a* and *b* increase. Within the calculation range, the 2D-MPHM’s maximum LE reaches as high as 12.8226, surpassing the majority of existing chaotic maps. Consequently, from the standpoint of LE, the 2D-MPHM demonstrates excellent chaotic performance and can fulfill the requirements of various engineering applications, such as image encryption.

### 2.3. Bifurcation and Trajectory Diagrams

**Figure 4:**
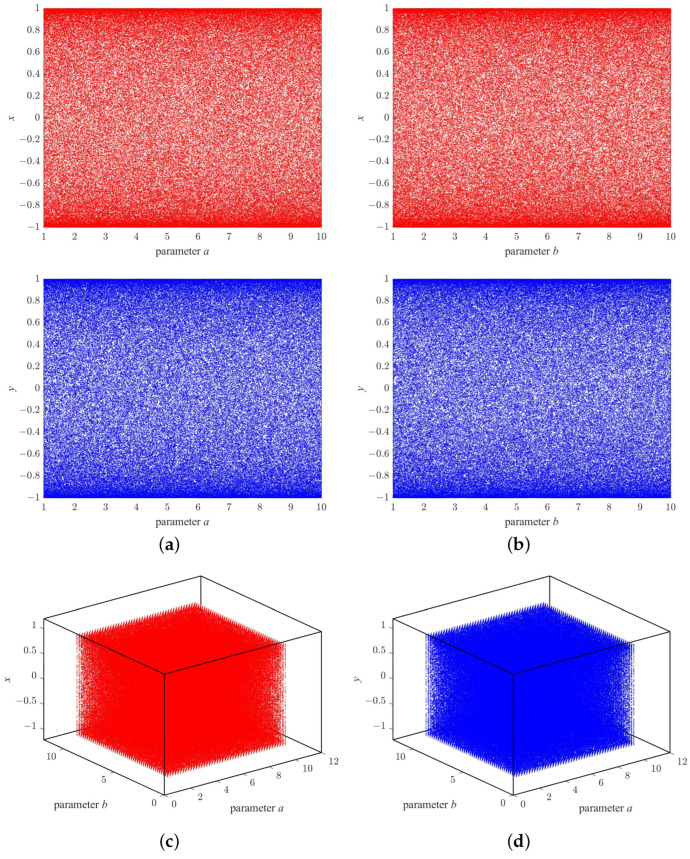
Six bifurcation diagrams for 2D-MPHM: (**a**) a=5; (**b**) b=8; (**c**,**d**) 3D bifurcation diagrams.

Bifurcation diagrams are commonly utilized to illustrate the chaotic behaviors of a system, enabling us to visually determine whether the system is in a state of chaos. To plot a bifurcation diagram, one or two specific control parameters of the system are examined, and the corresponding state values are calculated iteratively and presented on the diagram. If the state values exhibit a linear distribution, it indicates that the system is in a periodic state. Conversely, if the state values take on a planar distribution, it indicates that the system is in a chaotic state. Figure 4 presents the bifurcation diagrams of the 2D-MPHM that were obtained under three distinct conditions. These diagrams encompass the bifurcation diagram for the variable parameter a∈[1,10] and the bifurcation diagram for the variable parameter b∈[1,10], as well as the 3D bifurcation diagram when both *a* and *b* are variable parameters.

By examining these bifurcation diagrams, it becomes apparent that the 2D-MPHM consistently exhibits chaotic behavior across all parameter ranges, with its state values being uniformly scattered throughout the state space. This reveals that the 2D-MPHM possesses exceptional chaotic characteristics, making it highly suitable for a multitude of potential applications, particularly image encryption.

**Figure 5:**
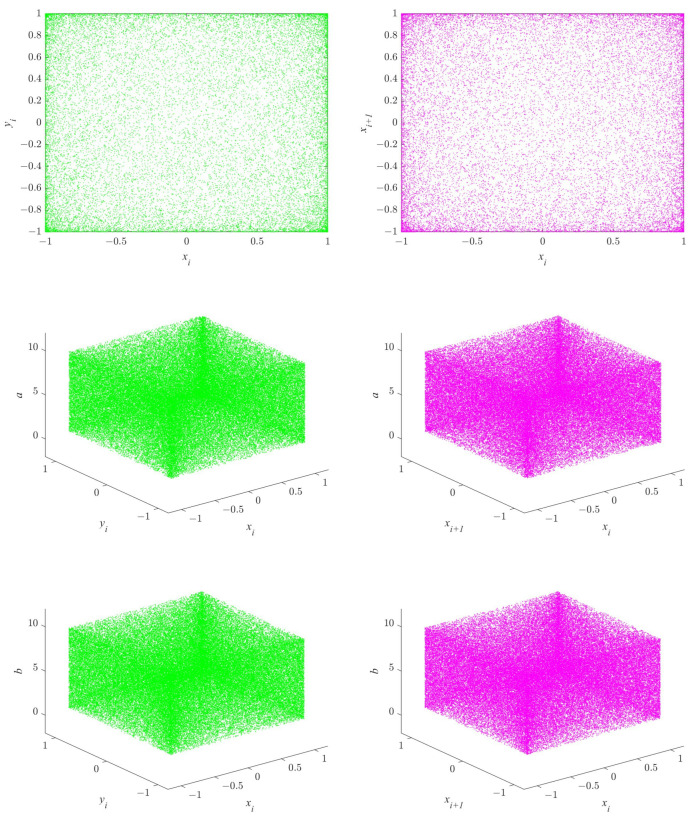
Six trajectory diagrams for 2D-MPHM: the first row is the trajectory diagram plotted when (a,b)=(5,8); the second row is trajectory diagram plotted when b=8; the third row is trajectory diagram plotted when a=5.

Additionally, we have also plotted the trajectory diagrams of the 2D-MPHM, which are presented in Figure 5. Upon observing each trajectory diagram, it becomes evident that the trajectory of the 2D-MPHM is highly uniformly in the phase plane and effectively occupies the entire state space. This once again confirms that the 2D-MPHM does possess excellent chaotic properties, making it an ideal choice for applications like image encryption.

### 2.4. Sample Entropy

**Figure 6:**
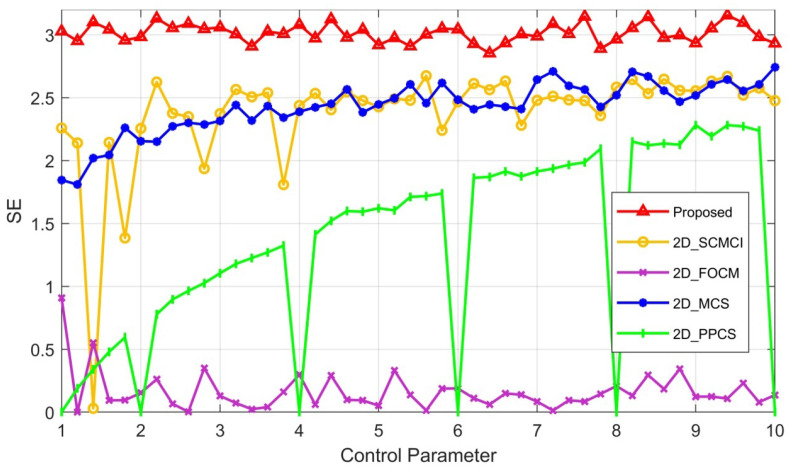
SE comparison for 2D-MPHM and four other newly proposed 2D maps.

Sample entropy (SE) characterizes the complexity of a time series by measuring the probability of generating new patterns in the signal. A higher probability of generating new patterns indicates a higher level of sequence complexity. For the chaotic sequence generated by a chaotic system, we can evaluate its complexity by calculating its SE. If the obtained SE value is large, it indicates that the self-similarity of the sequence is low and the complexity is high. We calculated the SE value of the chaotic sequence generated by the 2D-MPHM and compared it with those of four other newly reported chaotic maps. Figure 6 demonstrates the pertinent test results. As we can see, the SE values achieved by the 2D-MPHM are significantly higher than those of 2D-SCMCI [44], 2D-FOCM [45], 2D-MCS [46], and 2D-PPCS [47]. This indicates that when compared to these recent chaotic maps, the 2D-MPHM holds considerable advantages in terms of chaotic complexity.

### 2.5. Kolmogorov Entropy

**Figure 7:**
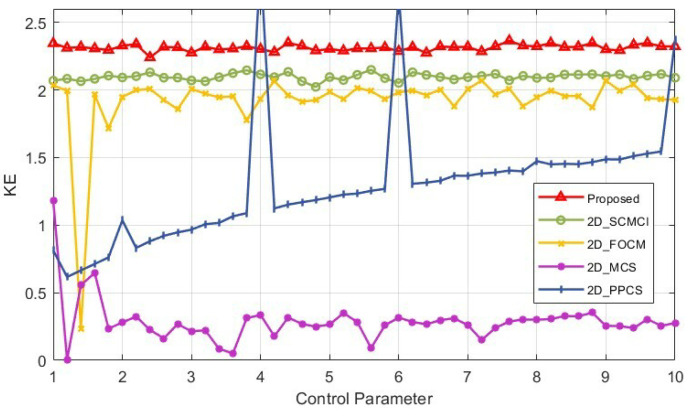
KE comparison for 2D-MPHM and four other newly proposed 2D maps.

Like SE, Kolmogorov entropy (KE) is often used by researchers to evaluate the chaotic properties of a system. Generally, the larger the KE value of a system, the higher the unpredictability and complexity of the system. Figure 7 shows the KE values of the 2D-MPHM and its comparison with those of the other four 2D chaotic maps. It can be seen from the figure that the 2D-MPHM has the highest KE value in most parameter ranges, and is stable at around 2.3. The results show that the 2D-MPHM has stable and excellent chaotic complexity.

## 3. Proposed MIEA-MPHM

**Figure 8:**
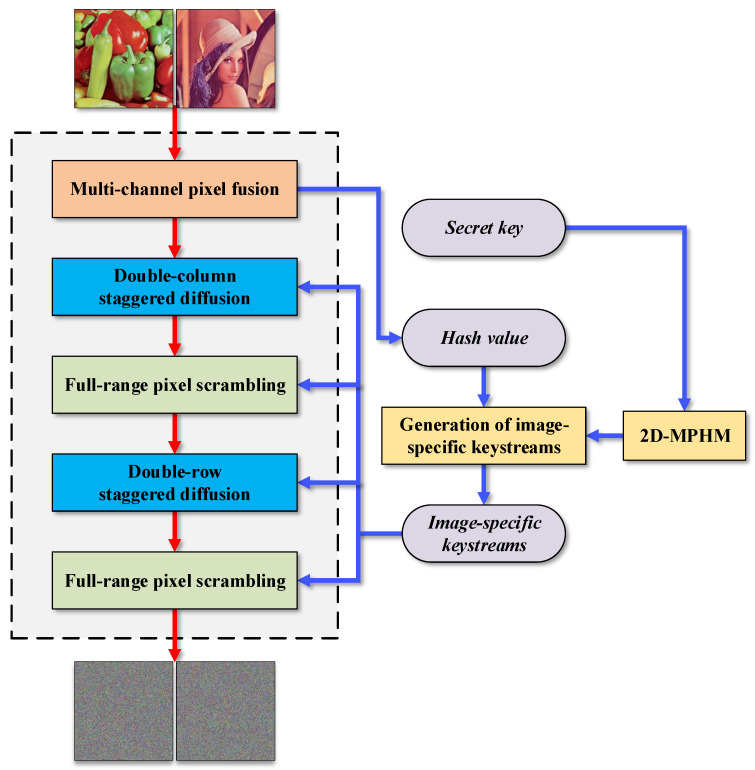
Encryption process of MIEA-MPHM.

To highlight the 2D-MPHM’s superiority in engineering applications and improve the security and efficiency of image encryption, we developed a highly efficient multi-channel image encryption algorithm called an MIEA-MPHM based on the 2D-MPHM. This encryption algorithm consists of seven encryption steps, namely ***the generation of an initial chaotic sequence***, ***multi-channel pixel fusion***, ***generation of image-specific keystreams***, and two rounds of ***double-vector (column or row) staggered diffusion*** and ***full-range pixel scrambling***. Figure 8 depicts the encryption process of the MIEA-MPHM. In the subsequent subsections, we will provide a comprehensive explanation of the entire encryption process for the MIEA-MPHM.

### 3.1. Generation of Initial Chaotic Sequence

For images that need to be encrypted and subsequently transmitted over a public channel, we assume that their maximum size is $H^{\left(M\right)}\times W^{\left(M\right)}$. In the MIEA-MPHM, we first utilize the proposed 2D-MPHM to generate an initial chaotic sequence. This chaotic sequence is then transformed into image-specific keystreams, thereby enhancing the plaintext sensitivity of the encryption process. Specifically, the process of generating the initial chaotic sequence is as follows:

- **Step 1**: Input the four components $\left\{x_{0},y_{0},a,b\right\}$ of the secret key $K=\{\gamma ,x_{0},y_{0},a,b\}$ into the 2D-MPHM as its initial states and control parameters.
- **Step 2**: Initialize the output chaotic sequence $Q^{\left(1\right)}$ with the length of
  (6) $$L=512+\gamma +2^{11}+2\times H^{\left(M\right)}\times W^{\left(M\right)}+H^{\left(M\right)}+W^{\left(M\right)}.$$
  This sequence will be used to save the resulting hyper-chaotic map state values.
- **Step 3**: Iterate the 2D-MPHM and sequentially save the two state values obtained for each iteration to $Q^{\left(1\right)}$; that is, for the *i*-th iteration, let $Q^{\left(1\right)}\left(2\times i-1\right)=x_{i}$ and $Q^{\left(1\right)}\left(2\times i\right)=y_{i}$.
- **Step 4**: Keep iterating the 2D-MPHM until all elements in $Q^{\left(1\right)}$ have changed to the state values of the 2D-MPHM.
- **Step 5**: Perform an interception operation on $Q^{\left(1\right)}$ and discard the first 512+γ elements to obtain the final initial chaotic sequence $Q^{\left(2\right)}$; that is, let
  (7) $$Q^{\left(2\right)}=Q^{\left(1\right)}\left(512+\gamma +1:L\right).$$

### 3.2. Multi-Channel Pixel Fusion

Currently, the vast majority of encryption algorithms encrypt images in units of bits, double bits (DNA bases), or pixels. This actually does not fully utilize the computing bandwidth of current mainstream 64-bit processors. Therefore, in order to more fully utilize the computing power of 64-bit processors, we perform pixel fusion on the input images. In this way, the calculation amount of subsequent encryption operations will be significantly reduced, thereby greatly promoting the improvement of encryption efficiency. First, we aggregate the input six grayscale images or two color images into a multi-channel image P of size H×W×6. Then, we perform multi-channel pixel fusion on the obtained P. Algorithm 1 shows the fusion process performed on P.
**Algorithm 1** Multi-channel pixel fusion algorithm.**Input:** The multi-channel image P with the size of H×W×6.  1:Initialize an all-zero matrix $C^{\left(1\right)}$ of size H×W;  2:**for** r=1 to 6 **do**  3:   $C^{\left(1\right)}=C^{\left(1\right)}+P\left(:,:,r\right)\times 2^{48-8\times r}$;  4:**end for****Output:** The fused image $C^{\left(1\right)}$.

### 3.3. Generation of Image-Specific Keystreams

Given the high input sensitivity of hash functions like SHA-256, numerous existing image encryption algorithms make use of them to enhance plaintext sensitivity. This helps in effectively defending against different types of plaintext attacks. However, these encryption algorithms directly employ the hash value of the input image as either the secret key or the input parameters of the chaotic system. Such approaches lead to practical issues, such as the requirement to recreate chaotic sequences or constantly change secret keys. In the MIEA-MPHM, we utilize the image hash value h to transform the initial chaotic sequence $Q^{\left(2\right)}$ into image-specific keystreams $S^{\left(1\right)}$, $S^{\left(2\right)}$, $S^{\left(3\right)}$, and $S^{\left(4\right)}$. In this way, it is possible to enhance plaintext sensitivity while circumventing potential practical problems. Specifically, the generation process of $S^{\left(1\right)}$, $S^{\left(2\right)}$, $S^{\left(3\right)}$, and $S^{\left(3\right)}$ is as follows:

- **Step 1**: Utilize the SHA-256 hash function to obtain the hash value h of the fused image $C^{\left(1\right)}$. The size of $C^{\left(1\right)}$ is H×W.
- **Step 2**: Further split h with the length of 256 bits into 8-bit bytes, which are $B_{1},B_{2},\cdots ,B_{32}$.
- **Step 3**: Sum these hash value bytes and then perform the modular operation to obtain
  (8) $$\sigma =(\left(\sum_{r=1}^{32}B_{r}\right)\;\mathrm{mod}\;2^{11})+1.$$
- **Step 4**: Leverage σ and the initial chaotic sequence $Q^{\left(2\right)}$ to obtain the image-specific keystreams $S^{\left(1\right)}$, $S^{\left(2\right)}$, $S^{\left(3\right)}$, and $S^{\left(4\right)}$:
  (9) $$S^{\left(1\right)}=\left\lfloor Q^{\left(2\right)}\left(\sigma +1:\sigma +W\right)\times 10^{15}\right\rfloor \;\mathrm{mod}\;16,$$
  (10) $$S^{\left(2\right)}=reshape\left(\left\lfloor Q^{\left(2\right)}\left(\sigma +W+1:\sigma +W+\theta^{\left(1\right)}\right)\times 10^{15}\right\rfloor \;\mathrm{mod}\;F\right),H,W),$$
  (11) $$S^{\left(3\right)}=\left\lfloor Q^{\left(2\right)}\left(\sigma +W+\theta^{\left(1\right)}+1:\sigma +\theta^{\left(1\right)}+\theta^{\left(2\right)}\right)\times 10^{15}\right\rfloor \;\mathrm{mod}\;16,$$
  (12) $$\;S^{\left(4\right)}=reshape\left(\left\lfloor Q^{\left(2\right)}\left(\sigma \;+\;\theta^{\left(1\right)}\;+\;\theta^{\left(2\right)}\;+\;1\;:\;\sigma \;+\;\theta^{\left(1\right)}\;+\;2\;\times \;\theta^{\left(1\right)}\right)\;\times \;10^{15}\right\rfloor \;\mathrm{mod}\;F\right),H,W),$$

where $\theta^{\left(1\right)}=H\times W$, $\theta^{\left(2\right)}=H+W$, and $F=2^{48}$. These image-specific keystreams will be used in subsequent encryption steps. Specifically, $S^{\left(1\right)}$ will be used in dual-column staggered diffusion; $S^{\left(2\right)}$ will be used in dual-column staggered diffusion and the first round of full-range pixel scrambling; $S^{\left(3\right)}$ will be used in dual-row staggered diffusion; and $S^{\left(4\right)}$ will be used in dual-row staggered diffusion and the second round of full-range pixel scrambling.

### 3.4. Double-Vector Staggered Diffusion

The diffusion process is crucial to guaranteeing the security of image encryption algorithms. However, numerous existing image encryption algorithms have been compromised as a result of relying on a single diffusion method. Thus, in the MIEA-MPHM, we meticulously devised and arranged two rounds of dual-vector staggered diffusion. In these two rounds of diffusion, the double-row staggered diffusion is the first one to be executed. The following steps outline its specific procedure:

- **Step 1**: Initialize an all-zero matrix $C^{\left(2\right)}$ with the size of H×W.
- **Step 2**: Conduct double-column diffusion on the first column of the fused image $C^{\left(1\right)}$ using modular addition operations:
  (13) $$\begin{array}{c} C^{\left(2\right)}\left(:,1\right)=(C^{\left(1\right)}\left(:,1\right)+S^{\left(1\right)}\left(2\right)\times C^{\left(1\right)}\left(:,N-1\right)+S^{\left(1\right)}\left(1\right)\times C^{\left(1\right)}\left(:,N\right)\\ +S^{\left(2\right)}\left(:,1\right))\;\mathrm{mod}\;F, \end{array}$$
  where $S^{\left(1\right)}$ and $S^{\left(2\right)}$ are the image-specific keystreams defined in Equations (9) and (10), and $F=2^{48}$.
- **Step 3**: Perform double-column diffusion on the second column of $C^{\left(1\right)}$ using XOR operations:
  (14) $$C^{\left(2\right)}\left(:,2\right)=C^{\left(1\right)}\left(:,2\right)⊕C^{\left(1\right)}\left(:,N\right)⊕C^{\left(2\right)}\left(:,1\right)⊕S^{\left(2\right)}\left(:,2\right).$$
- **Step 4**: Set i=2,3,⋯,N/2. For each *i*, conduct double-column diffusion on the (2×i−1)-th column and (2×i)-th column of $C^{\left(1\right)}$ in a staggered manner using modular addition and XOR operations:
  (15) $$\begin{array}{c} C^{\left(2\right)}\left(:,2\times i-1\right)=\;(C^{\left(1\right)}\left(:,2\times i-1\right)+S^{\left(1\right)}\left(2\times i-1\right)\times C^{\left(2\right)}\left(:,2\times i-3\right)\\ +S^{\left(1\right)}\left(2\times i\right)\times C^{\left(2\right)}\left(:,2\times i-2\right)+S^{\left(2\right)}\left(:,2\times i-1\right))\;\mathrm{mod}\;F, \end{array}$$
  (16) $$C^{\left(2\right)}\left(:,2\times i\right)=C^{\left(1\right)}\left(:,2\right)⊕C^{\left(2\right)}\left(:,2\times i-2\right)⊕C^{\left(2\right)}\left(:,2\times i-1\right)⊕S^{\left(2\right)}\left(:,2\times i\right),$$

The subsequent double-row staggered diffusion is essentially identical to the double-column staggered diffusion, with the difference being the change in the unit of diffusion from columns to rows. Furthermore, in the case of double-column staggered diffusion, the keystreams $S^{\left(1\right)}$ and $S^{\left(2\right)}$ are substituted with $S^{\left(3\right)}$ and $S^{\left(4\right)}$, respectively.

### 3.5. Full-Range Pixel Scrambling

As we know, plaintext attacks are the most threatening attack methods for image encryption algorithms. Many existing image encryption algorithms have been broken as they cannot effectively resist such attacks. To enhance the robustness of the MIEA-MPHM and prevent potential plaintext attacks, we incorporated a full-range pixel scrambling process after every round of staggered diffusion. Compared to common scrambling–diffusion structures, the MIEA-MPHM adopts a diffusion–scrambling structure. This design effectively thwarts attackers from employing chosen plaintext images with single pixel values to invalidate scrambling operations. Moveover, the MIEA-MPHM also reuses the key streams $S^{\left(2\right)}$ and $S^{\left(4\right)}$ utilized in the diffusion operations while performing scrambling operations. This reduces the length of the chaotic sequence that needs to be generated, thus promoting the improvement of encryption efficiency. Specifically, the steps for the first round of full-range pixel scrambling are as follows:

- **Step 1**: Initialize a matrix $C^{\left(3\right)}$ with the size of H×W, and let $C^{\left(3\right)}=C^{\left(2\right)}$.
- **Step 2**: Transform $S^{\left(2\right)}$ into the required row index matrix
  (17) $$\Phi^{\left(H\right)}=\left(S^{\left(2\right)}\;\mathrm{mod}\;H\right)+1.$$
- **Step 3**: Similarly, transform $S^{\left(4\right)}$ into the required column index matrix
  (18) $$\Phi^{\left(W\right)}=\left(S^{\left(4\right)}\;\mathrm{mod}\;W\right)+1.$$
- **Step 4**: For each row index α from 1 to *H*, repeat **Step 5** to **Step 6**.
- **Step 5**: For each column index β from 1 to *W*, repeat **Step 6**.
- **Step 6**: Swap $C^{\left(3\right)}\left(\alpha ,\beta \right)$ with $C^{\left(3\right)}\left(\Phi^{\left(H\right)}\left(\alpha ,\beta \right),\Phi^{\left(W\right)}\left(\alpha ,\beta \right)\right)$.

The subsequent second round of scrambling is basically the same as the first round of scrambling. The only difference is that the conversion method for row and column index matrices is slightly different. In the second round of scrambling,

(19) $$\Phi^{\prime (H)}=\left(S^{\left(4\right)}\;\mathrm{mod}\;H\right)+1$$

and

(20) $$\Phi^{\prime (W)}=\left(S^{\left(2\right)}\;\mathrm{mod}\;W\right)+1.$$

Since our MIEA-MPHM is a symmetrically structured image encryption algorithm, the decryption process is the reverse of the encryption process. To avoid unnecessary lengthiness, we omit the description of the decryption process here. Next, we will conduct a series of tests and analyses on the MIEA-MPHM to comprehensively evaluate its security and encryption performance.

## 4. Performance Tests and Analyses

With the aim of verifying and assessing its security and efficiency, we performed an extensive range of tests and analyses on the MIEA-MPHM. In our tests, the test images originate from two widely employed standard test databases, known as USC-SIPI and CVG-UGR. In addition, the hardware configurations utilized are Intel CPU E3-1231 v3 and 8 GB RAM, while the software configurations are Windows 10 and MATLAB R2017a. To ensure more objective assessments and analyses of the MIEA-MPHM’s performance, we employed randomly generated secret keys to conduct various tests. Moreover, to facilitate comparisons and demonstrate the encryption effects, we split the fused form of the ciphertext pixels into 8-bit pixels.

### 4.1. Visual Effects of Encryption and Decryption

**Figure 9:**
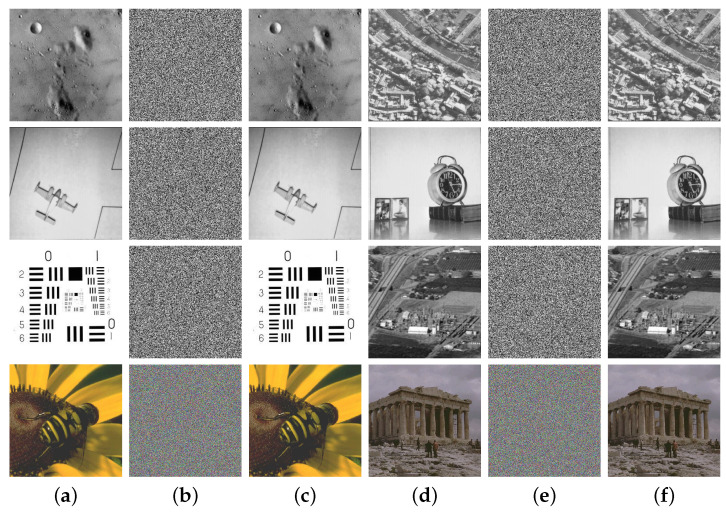
Visual effects of encryption and decryption: (**a**,**d**): six grayscale images and two color images; (**b**,**e**) encrypted ones generated by MIEA-MPHM; (**c**,**f**) decrypted ones generated by MIEA-MPHM.

For visual effects, a competent encryption algorithm should be able to entirely remove any meaningful information that can be perceived from the input image. On the other hand, the decrypted output image should fully restore all meaningful information. We conducted numerous tests on the MIEA-MPHM using randomly generated secret keys. In every test, the MIEA-MPHM could convert the input image into an unidentifiable noise image and restore the original image with no loss through decryption. Figure 9 shows the visual effects of our encryption and decryption tests. In the first encryption, six grayscale images (5.1.09, 5.1.10, 5.1.11, 5.1.12, 5.1.13, and 5.1.14) were encrypted simultaneously. In the second encryption, two color images (beeflowr and athens) were encrypted simultaneously. As can be seen, the ciphertext images generated by the MIEA-MPHM are entirely unrecognizable. Attackers cannot perceive any meaningful information from them. By utilizing the correct secret key, all authorized users can effortlessly obtain lossless decrypted images through decryption. Therefore, the visual effects of encryption and decryption provided by the MIEA-MPHM are in line with the requirements.

### 4.2. Key Space and Key Sensitivity

Among the various attacks against cryptosystems, brute-force attacks are the most common and easiest to implement. In general, brute-force attacks are typically leveraged to break a cryptosystem by exhaustively trying all possible keys within the key space. Therefore, in order to successfully resist different brute force attacks, a proposed image encryption algorithm should possess a key space of sufficient size. Currently, it is widely accepted that the key space should be equivalent to or larger than $2^{128}$. As described in Section 3.1, the secret key K of the MIEA-MPHM consists of five parts: γ, $x_{0}$, $y_{0}$, *a*, and *b*. Accordingly, one can easily determine the key space of the MIEA-MPHM, which is

(21) $$\phi^{\left(K\right)}=\phi^{\left(\gamma \right)}\times \phi^{\left(x_{0}\right)}\times \phi^{\left(y_{0}\right)}\times \phi^{\left(a\right)}\times \phi^{\left(b\right)}=3.3178\times 10^{65}\approx 2^{217}.$$

Since $\phi^{\left(K\right)}$ is much greater than $2^{128}$, the proposed MIEA-MPHM can effectively resist potential brute-force attacks.

**Figure 10:**
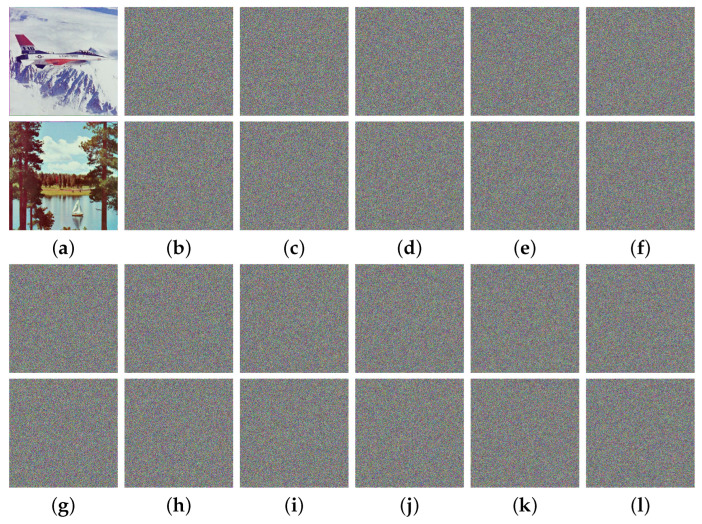
Key sensitivity test results for MIEA-MPHM: (**a**) 4.2.05 and 4.2.06; (**b**) ciphertext images generated with $\gamma^{\left(R\right)}=\gamma^{\left(R\right)}+1$; (**c**) generated with $x_{0}^{\left(R\right)}=x_{0}^{\left(R\right)}+10^{-15}$; (**d**) $y_{0}^{\left(R\right)}=y_{0}^{\left(R\right)}+10^{-15}$; (**e**) $a^{\left(R\right)}=a^{\left(R\right)}+10^{-15}$; (**f**) $b^{\left(R\right)}=b^{\left(R\right)}+10^{-15}$; (**g**) original ciphertext images of 4.2.05 and 4.2.06; (**h**) differences between (**b**,**g**); (**i**) differences between (**c**,**g**); (**j**) differences between (**d**,**g**); (**k**) differences between (**e**,**g**); (**l**) differences between (**f**,**g**).

Based on prior cryptography research, it is suggested that the relationship between the secret key and the ciphertext should be as complex as possible. This implies that even if the key changes only slightly, the ciphertext should undergo significant changes. In simpler terms, a suggested image encryption algorithm must exhibit a high degree of sensitivity toward key alterations. Several tests were conducted to evaluate the MIEA-MPHM’s key sensitivity. One of these tests is represented in Figure 10, which shows the corresponding results. In this test, we generated a random secret key

(22) $$K^{\left(R\right)}=\left\{\begin{array}{c} \gamma^{\left(R\right)}=484,\\ x_{0}^{\left(R\right)}=0.338090773927741,\\ y_{0}^{\left(R\right)}=0.075162771765254,\\ a^{\left(R\right)}=0.194490737784561,\\ b^{\left(R\right)}=0.051605090236889, \end{array}\right.$$

and adopted it to encrypt the test images 4.2.05 and 4.2.06. Afterwards, we made minimal changes to each component of $K^{\left(R\right)}$, resulting in five new secret keys. Finally, we encrypted the same test images using these five secret keys and generated difference images between the new ciphertext images and the original ones. Upon observing Figure 10, it becomes evident that even the slightest alteration in any component will result in completely different ciphertext images. Moreover, if these changes themselves are presented in the form of images, they are also highly unrecognizable quasi-random images. Hence, the MIEA-MPHM has superior key sensitivity and can effectively resist potential attacks related to the statistical relationship between the secret key and ciphertexts.

### 4.3. Plaintext Sensitivity

**Figure 11:**
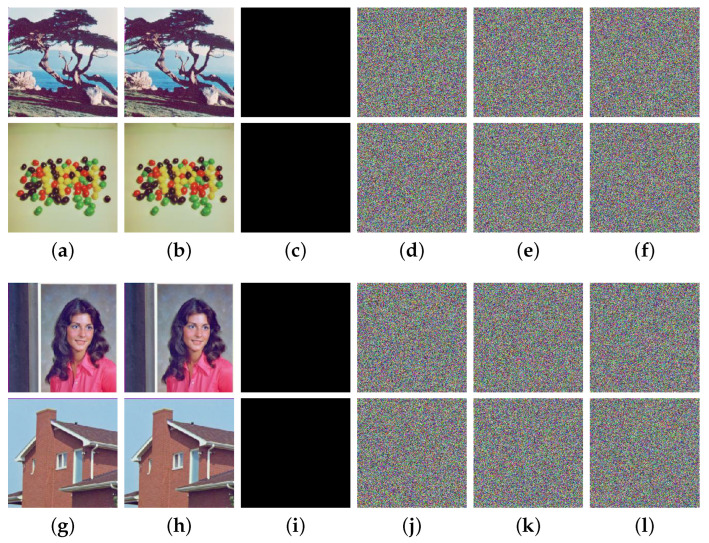
Plaintext sensitivity test results for MIEA-MPHM: (**a**) the first set of input images 4.1.06 and 4.1.08; (**b**) one pixel bit located at (1,1,1) in 4.1.06 was changed; (**c**) difference between (**a**,**b**); (**d**) ciphertext of (**a**); (**e**) ciphertext of (**b**); (**f**) difference between (**d**,**e**); (**g**) the second set of input images 4.1.04 and 4.1.05; (**h**) one pixel bit located at (256,256,3) in 4.1.05 was changed; (**i**) difference between (**g**,**h**); (**j**) ciphertext of (**g**); (**k**) ciphertext of (**h**); (**l**) difference between (**j**,**k**).

To effectively withstand a range of differential attacks, particularly plaintext attacks, a robust cryptosystem should possess a remarkably high level of sensitivity toward plaintext. This means that even if the plaintext undergoes only minimal changes, the corresponding ciphertext should change significantly. In order to evaluate the plaintext sensitivity of the MIEA-MPHM, we performed two minimum modifications on two sets of color image inputs simultaneously. For the first modification, we modified the lowest bit of the first pixel, which is located at (1,1) of the red channel in 4.1.06. In the second modification, the lowest bit of the last pixel was modified, which is located at (256,256) of the blue channel of 4.1.05. Subsequently, we encrypted the four sets of inputs to analyze the ciphertext changes caused by the modifications. As can be observed from Figure 11, for each modification, even though only one channel experiences a minimal change of one bit, all corresponding ciphertext images undergo complete alteration. Moreover, these significant changes are not influenced by the location of the plaintext modification. Thus, our MIEA-MPHM exhibits excellent sensitivity toward plaintext.

**Figure 12:**
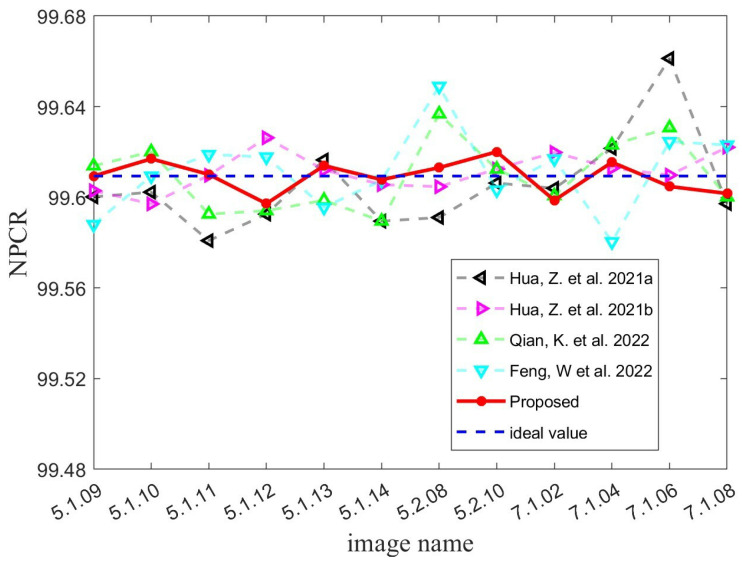
NPCR test results of MIEA-MPHM and four state-of-the-art algorithms [48,49,50,51].

**Figure 13:**
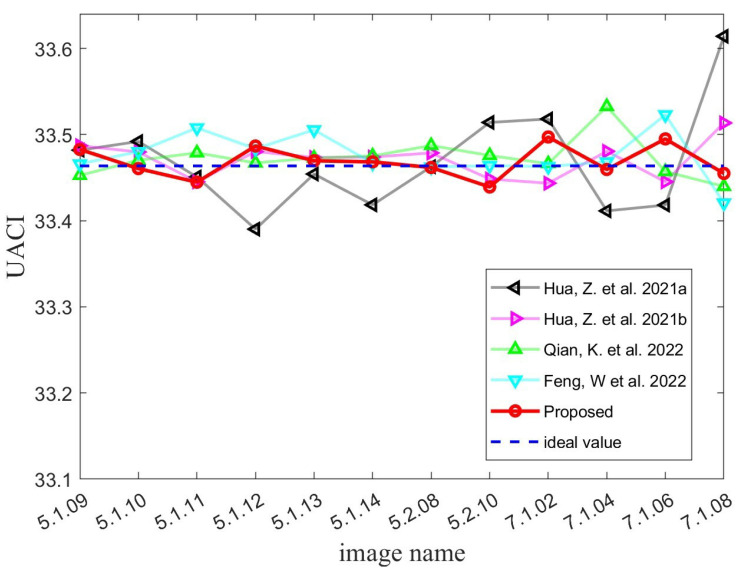
UACI test results of MIEA-MPHM and four state-of-the-art algorithms [48,49,50,51].

Additionally, in order to further emphasize the MIEA-MPHM’s outstanding performance in plaintext sensitivity, we have also conducted a multitude of quantitative analyses utilizing two widely adopted indices. These two indices are the number of pixels change rate (NPCR) and the unified average changing intensity (UACI). For the original ciphertext image $C_{1}$ and the changed ciphertext image $C_{2}$, one can use the following definitions to calculate the NPCR and UACI values between them:

(23) $$NPCR\left(C_{1},C_{2}\right)=\sum_{m=1}^{M}\sum_{n=1}^{N}D(m,n)/M\times N\times 100\%,$$

(24) $$UACI\left(C_{1},C_{2}\right)=\sum_{m=1}^{M}\sum_{n=1}^{N}\left|C_{1}\left(m,n\right)-C_{2}\left(m,n\right)\right|/\left(255\times M\times N\right)\times 100\%,$$

where *M* indicates the height of the two images and *N* represents the width of them. D(m,n) stands for the difference value between $C_{1}\left(m,n\right)$ and $C_{2}\left(m,n\right)$. When $C_{1}\left(m,n\right)=C_{2}\left(m,n\right)$, D(m,n)=0; otherwise, D(m,n)=1. According to Equations (23) and (24), one can calculate the NPCR and UACI values between a given ciphertext image and a random image to be 99.6094% and 33.4635%, respectively. Therefore, when it comes to plaintext sensitivity tests, the ideal values of NPCR and UACI that an image encryption algorithm can achieve are 99.6094% and 33.4635%, respectively. Table 1 and Table 2 present the test results that we have obtained. As can be seen, the average values obtained by all five algorithms are very close to the ideal values. This demonstrates that all algorithms exhibit good plaintext sensitivity. Significantly, the MIEA-MPHM exhibits two test averages that are closest to the ideal values of 99.6094% and 33.4635%. Moreover, the MIEA-MPHM’s test results are also the most stable. To highlight the advantages of the MIEA-MPHM more intuitively, we plotted the test results of all five algorithms into two diagrams, as shown in Figure 12 and Figure 13. Please note that in Figure 12 and Figure 13, black line represents the image encryption algorithm proposed in [48], violet line represents the image encryption algorithm proposed in [49], green line represents the image encryption algorithm proposed in [50], and blue line represents the image encryption algorithm proposed in [51].Therefore, the MIEA-MPHM has first-class plaintext sensitivity and can resist various potential differential attacks.

### 4.4. Information Entropy

Since it can reflect the randomness of information sources, information entropy (IE) is commonly utilized for evaluating the randomness and distribution uniformity of ciphertext pixels. For an image with a pixel depth of 8 bits, one can employ the following definition to calculate its IE value:

(25) $$IE\left(S\right)=-\sum_{j=1}^{q}\rho (S_{j})\log_{2}\rho (S_{j}),$$

where $S=\{S_{1},S_{2},\cdots S_{q}\}$, $\rho (S_{j})$ denotes the probability of $S_{j}$. Generally, for ciphertext images that need to be evaluated, higher IE values mean higher pixel randomness and distribution uniformity. According to Equation (25), the ideal IE value of a ciphertext image can be ascertained as 8. We encrypted many test images and calculated the IE value of the ciphertext images generated by the MIEA-MPHM. The relevant test results are shown in Table 3. Compared to ciphertext images, original images typically have smaller IE values. The IE values of all ciphertext images generated by the MIEA-MPHM are extremely close to 8. This indicates that the pixels in these ciphertext images are highly random and their distribution is remarkably uniform.

Furthermore, we also performed comparative experiments on IE. As indicated in Table 4, the MIEA-MPHM attains a higher IE value in contrast to the other eight latest encryption algorithms. This demonstrates that the MIEA-MPHM possesses distinct advantages in terms of IE, enabling it to produce ciphertext images with optimal randomness.

### 4.5. Pixel Distribution

**Figure 14:**
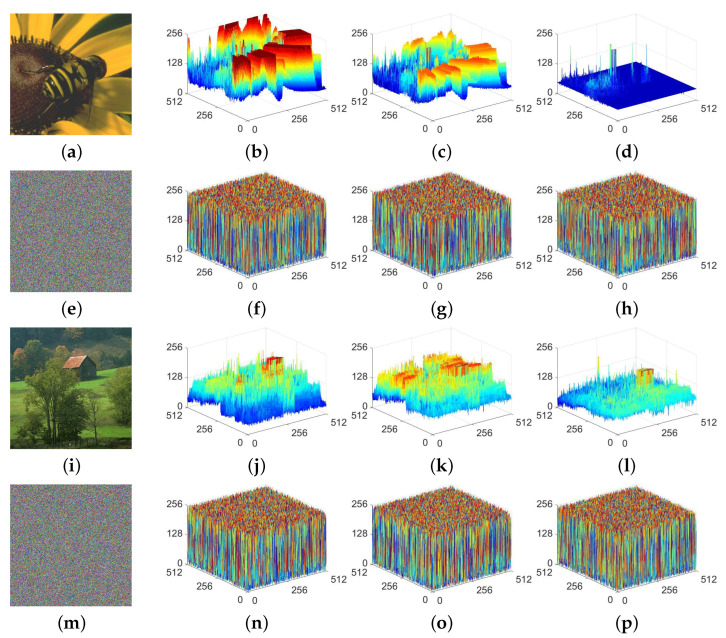
Three-dimensional pixel distribution diagrams of input images and their ciphertext images: (**a**) beeflowr; (**b**–**d**) are three diagrams for R, G, and B channels of (**a**); (**e**) ciphertext of (**a**); (**f**–**h**) are diagrams for (**e**); (**i**) barnfall; (**j**–**l**) are diagrams for (**i**); (**m**) ciphertext of (**i**); (**n**–**p**) are diagrams for (**m**).

It is common for natural images to have highly prominent distribution characteristics. Accordingly, a proposed encryption algorithm should be capable of effectively eliminating these characteristics, thus preventing any leakage of information. To assess the MIEA-MPHM’s performance in pixel distribution, we simultaneously encrypted two color images using a random secret key. Subsequently, we further plotted 3D pixel distribution diagrams for the input images and the resulting ciphertext images. All these pixel distribution diagrams are provided in Figure 14.

By observing, we can determine that the pixel distribution in each channel of these two color images is highly uneven. Nonetheless, in the corresponding encrypted images output by the MIEA-MPHM, these prominent distribution features have been entirely eradicated. The pixel distribution of each channel becomes highly uniform. Therefore, the MIEA-MPHM exhibits excellent performance in terms of pixel distribution, which helps to prevent attackers from exploiting the distribution characteristics of ciphertext pixels for launching attacks.

### 4.6. Correlation Analysis

**Figure 15:**
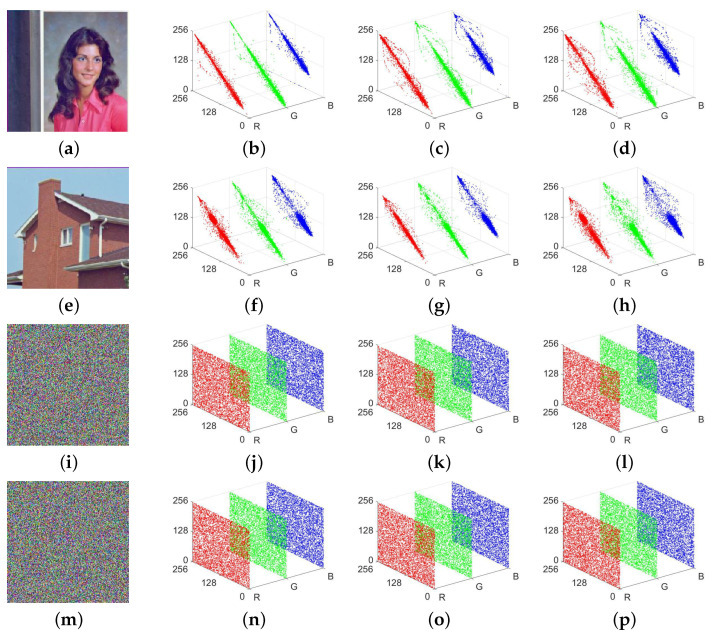
Pixel correlation analysis diagrams of input images and their ciphertext counterparts: (**a**) 4.1.04; (**b**) is the diagram for (**a**) in horizontal direction; (**c**) is the diagram for (**a**) in vertical direction; (**d**) is the diagram for (**a**) in diagonal direction; (**e**) 4.1.05; (**f**–**h**) are three diagrams for (**e**) in three directions; (**i**) ciphertext of 4.1.04; (**j**–**l**) are three diagrams for (**i**); (**m**) ciphertext of 4.1.05; (**n**–**p**) are three diagrams for (**m**).

The strong correlation between adjacent pixels is one of the distinctive features of natural images. To counter potential attacks by adversaries who target this feature specifically, a proficient encryption algorithm must be capable of effectively minimizing the correlation between pixels. For the purpose of evaluating the MIEA-MPHM’s performance in decreasing pixel correlation, we encrypted two test images 4.1.04 and 4.1.05 simultaneously with a secret key that was randomly generated. After obtaining corresponding ciphertext images, we further drew correlation analysis diagrams for the original images and their ciphertext counterparts. By observing Figure 15, one can see that in the two original images, for each direction (horizontal, vertical, and diagonal) of each channel (red, green, and blue), adjacent pixels show a strong correlation close to 1. In contrast, the ciphertext images generated by the MIEA-MPHM exhibit complete dissimilarity. We cannot detect any correlation traits in their analysis diagrams.

Additionally, we conducted many quantitative analyses on the effectiveness of the MIEA-MPHM in reducing pixel correlation. The indicator utilized in our analyses is the correlation coefficient (CC). It is also a widely used indicator for security assessment, and its mathematical definition is as follows:

(26) $$CC=\frac{E(\left(V_{m}-E\left(V_{m}\right)\right)\left(V_{n}-E\left(V_{n}\right)\right)}{\sqrt{D\left(V_{m}\right)D\left(V_{n}\right)}},$$

where $V_{m}$ and $V_{n}$ denote pixel values, $E(V_{m})$ and $E(V_{n})$ stand for expectations, and $D(V_{m})$ and $D(A_{n})$ indicate variances. The relevant analysis results are provided in Table 5. As can be seen, the CC values of two original images are significantly high in every direction of every channel. Conversely, in the ciphertext images generated by the MIEA-MPHM, all pixel correlations are effectively removed. The corresponding CC values plummet to extremely low values, close to 0. This indicates that the MIEA-MPHM features excellent performance in reducing pixel correlation.

### 4.7. Robustness Analysis

**Figure 16:**
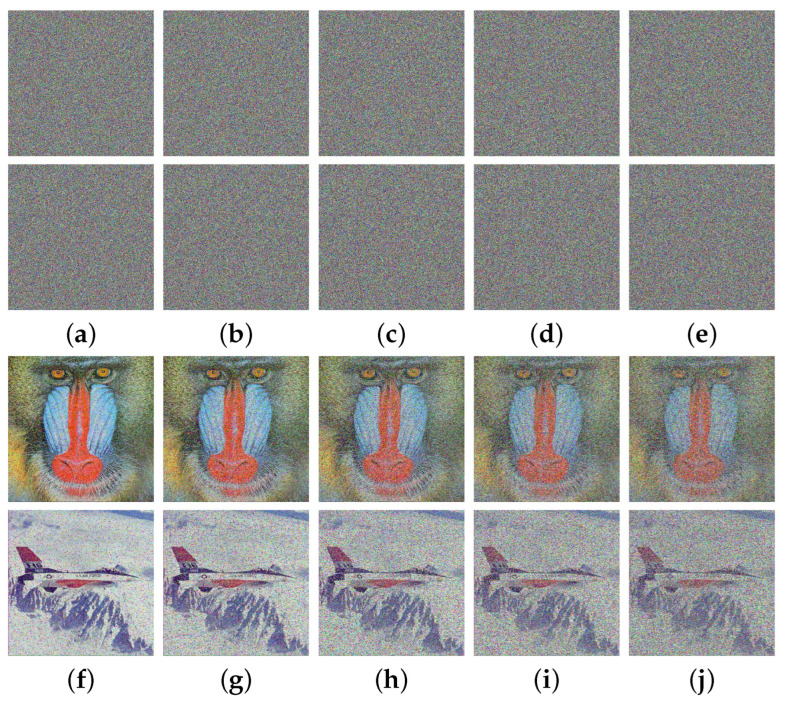
Robustness analysis on noise for MIEA-MPHM: (**a**) ciphertext images with salt-and-pepper noise of intensity 0.02; (**b**) noise of intensity 0.04; (**c**) noise of intensity 0.06; (**d**) noise of intensity 0.08; (**e**) noise of intensity 0.10; (**f**–**j**) are decrypted images of (**a**–**e**).

In today’s complex application environment, ciphertext images may experience data loss during transmission or storage. Consequently, a proposed encryption algorithm should be robust enough to endure significant data loss. Two rounds of tests were conducted to evaluate and analyze the robustness of the MIEA-MPHM. In the first round of testing, we intentionally added five different intensities of salt-and-pepper noise to the ciphertext images. Afterwards, we decrypted the ciphertext images that were impacted by noise. The pertinent test results are illustrated in Figure 16. As can be seen, the decrypted images remain essentially unaffected when the noise intensity is low. As the intensity of noise increases, the decrypted image progressively becomes blurry. This implies that the proportion of useful information retained in the decrypted image depends on the level of noise intensity. If the intensity of noise added to the ciphertext image is higher, then less information will be retained, resulting in a higher degree of blurriness in the decrypted image. However, despite the noise intensity being as high as 0.10, the MIEA-MPHM can still effectively restore the vast majority of meaningful visual information presented in the original images.

**Figure 17:**
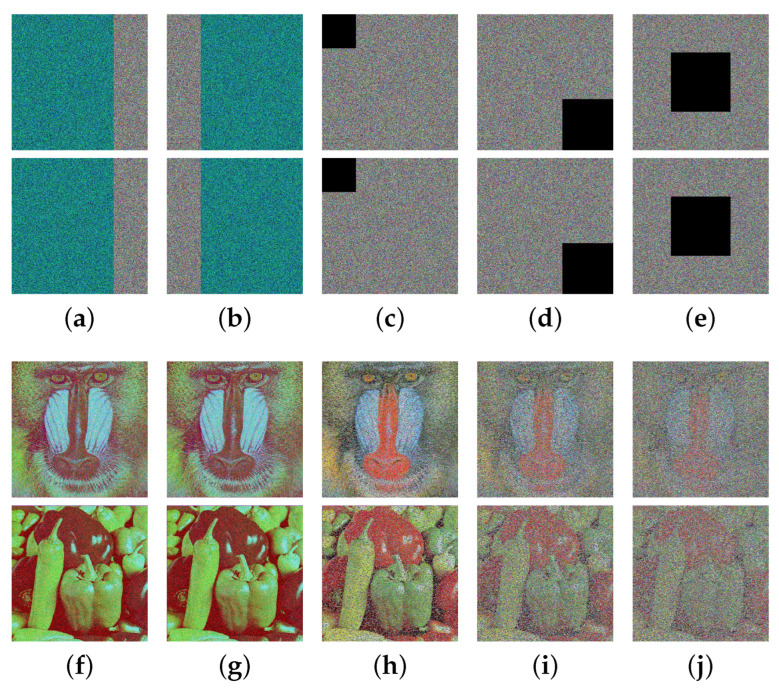
Robustness analysis on data loss for MIEA-MPHM: (**a**) 512×384 pixels missing in red channel; (**b**) 512×384 pixels missing in blue channel; (**c**) 128×128 pixels missing in all channels; (**d**) 192×192×6 pixels missing; (**e**) 224×224×6 pixels missing; (**f**–**j**) are decrypted images of (**a**–**e**).

In the second round of testing, we intentionally removed certain ciphertext pixels, as illustrated in Figure 17. Through observation, one can see that when significant data losses occur in a single channel, the visual quality of the decrypted images is hardly affected. And, when all channels simultaneously suffer data losses, the decrypted images become blurry. Similarly, the proportion of useful information retained in the decrypted image depends on the number of missing ciphertext pixels. If the number of missing ciphertext pixels increases, less information will be retained, leading to a more blurred decrypted image. Significantly, when the data loss is as high as about 20%, the decrypted images can still retain most useful visual information. In summary, the MIEA-MPHM exhibits exceptional robustness and can satisfactorily restore the original images in cases where the ciphertext images experience considerable data losses.

### 4.8. Encryption Efficiency

As we know, aside from enhancing security, one of the primary motivations for developing new image encryption algorithms is to attain higher encryption rates. In our proposed MIEA-MPHM, we have incorporated several targeted designs to maximize the encryption rate. Firstly, a 2D-MPHM is a hyper-chaotic map with a simple structure, so it can efficiently generate chaotic sequences. Secondly, the MIEA-MPHM optimizes the use of the plaintext hash value. The regeneration of chaotic sequences is no longer necessary for ensuring plaintext sensitivity. Finally, and more importantly, the MIEA-MPHM has introduced an innovative pixel fusion technique, and this technique can more fully utilize the computing bandwidth of today’s mainstream 64-bit processors.

To showcase the MIEA-MPHM’s substantial advantages in encryption efficiency, we conducted comprehensive tests and compared its test results with those of six recent algorithms. By observing Table 6, we can find that the average encryption rate of the MIEA-MPHM is significantly higher than that of other algorithms. Among all of these algorithms, the average encryption rate of the MIEA-MPHM is nearly three times higher than the second-ranked algorithm and nearly eighty times higher than the slowest algorithm. To encrypt an image with a size of 1024×1024, the MIEA-MPHM only takes 0.1036 s on average, and its average encryption rate is as high as 87.2798 Mbps. Hence, when it comes to encryption efficiency, our proposed MIEA-MPHM offers greater practicality and can better fulfill the requirements of diverse practical applications.

## 5. Conclusions

To enrich the dynamical characteristics and increase the complexity, a 2D polynomial hyper-chaotic map known as a 2D-MPHM was created in this study using the discrete TiO${}_{2}$ memristor model. The map’s Lyapunov exponents, bifurcation diagrams, trajectory diagrams, sample entropy, and Kolmogorov entropy were calculated numerically. The experimental results show that this map has excellent chaotic performance, including a broad hyper-chaotic range, a uniform trajectory distribution, and a fast trajectory divergence rate. To emphasize the 2D-MPHM’s superiority in engineering applications and improve the security and efficiency of image encryption, we developed a highly efficient multi-channel image encryption algorithm named an MIEA-MPHM based on the 2D-MPHM.

In the MIEA-MPHM, we performed pixel fusion on the input images to better make use of the processing power of 64-bit processors. This significantly cut down on the number of calculations required for subsequent encryption operations, thereby greatly facilitating the improvement of encryption efficiency. The SHA-256 hash function was utilized to transform the initial chaotic sequence into image-specific keystreams. In this way, it is possible to enhance plaintext sensitivity while circumventing potential practical problems. To enhance the robustness of the MIEA-MPHM and prevent potential plaintext attacks, we incorporated a full-range pixel scrambling process after every round of staggered diffusion. Compared to common scrambling–diffusion structures, the MIEA-MPHM adopts a diffusion–scrambling structure. This design effectively thwarts attackers from employing chosen plaintext images with single pixel values to invalidate scrambling operations. Based on extensive tests and analyses, the MIEA-MPHM exhibits outstanding security, surpassing that of latest leading image encryption algorithms. Remarkably, the MIEA-MPHM also has an extremely obvious efficiency advantage, with an average encryption rate of up to 87.2798 Mbps. Consequently, the MIEA-MPHM is able to meet the needs of potential applications better than most existing image encryption algorithms.

Overall, our work possesses scientific novelty in two aspects. Firstly, we designed a 2D discrete hyper-chaotic map with exponential-form parameters and a cross-coupling architecture. Further, we optimized it by incorporating two memristors and thus obtained the final 2D-MPHM. Our proposed 2D-MPHM not only features a simple structure, but it also exhibits excellent chaotic performance, rendering it suitable for various engineering applications, including image encryption. Secondly, we developed a secure and efficient multi-channel image encryption algorithm by introducing several novel designs. These designs include a versatile encryption method capable of simultaneously encrypting multiple grayscale or color images, pixel fusion to significantly reduce encryption computation, and new permutation and diffusion techniques that ensure security while enhancing efficiency. In the future, we will strive to utilize memristors for constructing chaotic systems with improved performance. Concurrently, we will explore the potential of applying a 2D-MPHM to various fields, including neural network optimization and image compression.

## Figures and Tables

**Table 1:** NPCR test results of MIEA-MPHM and four state-of-the-art algorithms.

Size	Name	MIEA-MPHM	[48]	[49]	[50]	[51]
256×256	5.1.09	99.6094	99.6002	99.6029	99.6139	99.5880
	5.1.10	99.6170	99.6023	99.5971	99.6201	99.6094
	5.1.11	99.6101	99.5809	99.6097	99.5926	99.6189
	5.1.12	99.5972	99.5926	99.6263	99.5941	99.6178
	5.1.13	99.6140	99.6165	99.6121	99.5987	99.5956
	5.1.14	99.6078	99.5895	99.6056	99.5895	99.6075
512×512	5.2.08	99.6131	99.5911	99.6047	99.6368	99.6490
	5.2.10	99.6201	99.6063	99.6125	99.6124	99.6033
	7.1.02	99.5987	99.6040	99.6198	99.6007	99.6170
	7.1.04	99.6155	99.6216	99.6129	99.6231	99.5804
	7.1.06	99.6048	99.6613	99.6098	99.6307	99.6246
	7.1.08	99.6017	99.5972	99.6221	99.6002	99.6231
	Average	99.6091	99.6053	99.6113	99.6090	99.6112
	Std. Dev.	0.0073	0.0210	0.0084	0.0164	0.0183

**Table 2:** UACI test results of MIEA-MPHM and four state-of-the-art algorithms.

Size	Name	MIEA-MPHM	[48]	[49]	[50]	[51]
256×256	5.1.09	33.4827	33.4818	33.4870	33.4525	33.4658
	5.1.10	33.4604	33.4919	33.4797	33.4701	33.4801
	5.1.11	33.4446	33.4507	33.4452	33.4787	33.5077
	5.1.12	33.4866	33.3901	33.4802	33.4670	33.4835
	5.1.13	33.4695	33.4541	33.4733	33.4727	33.5054
	5.1.14	33.4682	33.4183	33.4738	33.4751	33.4667
512×512	5.2.08	33.4617	33.4626	33.4786	33.4872	33.4622
	5.2.10	33.4391	33.5140	33.4483	33.4756	33.4639
	7.1.02	33.4971	33.5180	33.4434	33.4661	33.4628
	7.1.04	33.4594	33.4113	33.4805	33.5325	33.4683
	7.1.06	33.4949	33.4181	33.4451	33.4569	33.5229
	7.1.08	33.4548	33.6140	33.5134	33.4397	33.4205
	Average	33.4683	33.4687	33.4707	33.4747	33.4758
	Std. Dev.	0.0187	0.0614	0.0213	0.0228	0.0269

**Table 3:** IE test results of MIEA-MPHM.

Size	Name	Original	Ciphertext
256×256	5.1.09	6.7093	7.9978
	5.1.10	7.3118	7.9978
	5.1.11	6.4523	7.9980
	5.1.12	6.7057	7.9978
	5.1.13	1.5483	7.9979
	5.1.14	7.3424	7.9978
512×512	5.2.08	7.2010	7.9994
	5.2.10	5.7056	7.9994
	7.1.02	4.0045	7.9993
	7.1.04	6.1074	7.9993
	7.1.06	6.6953	7.9993
	7.1.08	5.0534	7.9994

**Table 4:** IE values of MIEA-MPHM and other encryption algorithms.

Algorithm	IE
MIEA-MPHM	7.9994
[48]	7.9992
[50]	7.9992
[51]	7.9993
[52]	7.9993
[53]	7.9993
[54]	7.9993
[55]	7.9976
[56]	7.9984

**Table 5:** Obtained CC values for MIEA-MPHM.

Size	Name	Original			Ciphertext		
		**H**	**V**	**D**	**H**	**V**	**D**
256×256	5.1.09	0.9407	0.9005	0.9064	0.0036	−0.0005	0.0008
	5.1.10	0.8589	0.9068	0.8144	0.0006	−0.0026	0.0010
	5.1.11	0.9414	0.9519	0.8928	0.0038	0.0029	0.0013
	5.1.12	0.9697	0.9512	0.9379	−0.0015	−0.0027	−0.0015
	5.1.13	0.8722	0.8741	0.7587	−0.0009	−0.0014	0.0029
	5.1.14	0.8963	0.9478	0.8445	−0.0024	0.0012	0.0019
512×512	5.2.08	0.9053	0.9465	0.8562	0.0024	−0.0023	−0.0003
	5.2.10	0.9276	0.9384	0.9018	−0.0010	0.0011	0.0020
	7.1.02	0.9558	0.9420	0.9007	0.0012	−0.0027	−0.0001
	7.1.04	0.9650	0.9785	0.9580	0.0017	0.0010	−0.0007
	7.1.06	0.9106	0.9424	0.8806	−0.0014	0.0004	0.0022
	7.1.08	0.9277	0.9594	0.9222	0.0016	−0.0017	0.0011

**Table 6:** Encryption times (rates) of MIEA-MPHM and six recent algorithms.

Algorithm	256×256	512×512	1024×1024	Average
[50]	0.4341 s	1.7586 s	7.1223 s	–
	(1.1518 Mbps)	(1.1373 Mbps)	(1.1232 Mbps)	(1.1374 Mbps)
[52]	0.0768 s	0.3213 s	1.3806 s	–
	(6.5104 Mbps)	(6.2247 Mbps)	(5.7946 Mbps)	(6.1766 Mbps)
[53]	0.0203 s	0.0878 s	0.3755 s	–
	(24.6305 Mbps)	(22.7790 Mbps)	(21.3049 Mbps)	(22.9048 Mbps)
[55]	0.1524 s	0.6313 s	2.5712 s	–
	(3.2808 Mbps)	(3.1681 Mbps)	(3.1114 Mbps)	(3.1868 Mbps)
[57]	0.0915 s	0.4088 s	2.0314 s	–
	(5.4645 Mbps)	(4.8924 Mbps)	(3.9382 Mbps)	(4.7650 Mbps)
[58]	0.0800 s	0.4842 s	2.2848 s	–
	(6.2500 Mbps)	(4.1305 Mbps)	(3.5014 Mbps)	(4.6273 Mbps)
**MIEA-MPHM**	**0.0051 s**	**0.0231 s**	**0.1036 s**	–
	(**98.0392 Mbps**)	(**86.5801 Mbps**)	(**77.2201 Mbps**)	(**87.2798 Mbps**)

## Data Availability

Data will be made available on request.

## Abbreviations

The following abbreviations are used in this paper:

2D-MPHM	2D memristor-enhanced polynomial hyper-chaotic map
MIEA-MPHM	Multi-channel image encryption algorithm based on 2D-MPHM
PWL	Piecewise linear
DNA	Deoxyribonucleic acid
LE	Lyapunov exponent
SE	Sample entropy
KE	Kolmogorov entropy
NPCR	Number of pixels change rate
UACI	Unified average changing intensity
IE	Information entropy
CC	Correlation coefficient
